# Single Nucleotide Polymorphism of SREBF-1 Gene Associated with an Increased Risk of Endometrial Cancer in Chinese Women

**DOI:** 10.1371/journal.pone.0090491

**Published:** 2014-03-10

**Authors:** Chun-Ping Qiu, Qing-Tao Lv, Samina Dongol, Chenguang Wang, Jie Jiang

**Affiliations:** 1 Department of Obstetrics and Gynecology, Qilu Hospital, Shandong University, Jinan, P R China; 2 Department of Pharmaceutical Chemistry, Shandong University of Traditional Chinese Medicine, Jinan, P R China; 3 Departments of Cancer Biology, Stem Cell Biology and Regenerative Medicine, Kimmel Cancer Center, Thomas Jefferson University, Philadelphia, Pennsylvania, United States of America; CHA University, Republic of Korea

## Abstract

**Aim:**

Elevated levels of sterol regulatory element-binding protein-1 (SREBP-1) have been found in endometrial cancer (EC), suggesting that it is essential to the development of EC. Obesity and diabetes have been established as known risk factors of EC, while SREBF-1 gene polymorphisms have also been found to be associated with obesity and type II diabetes. Therefore, we hypothesize that single nucleotide polymorphism (SNP) in SREBF-1 gene may be associated with increased risk of EC.

**Method:**

We analyzed the sequence of SREBF-1 in tissue samples from 30 EC cases and 6 benign controls using high throughput method. Based on the primary results, we selected one SNP (rs2297508) as a genetic marker to conduct a hospital-based case-control study with 139 EC cases and 129 benign controls. The samples were examined under the microscope to determine their histopathology prior to the SNP analysis using RT-PCR.

**Results:**

Through sequence analysis, we found 10 SNPs of SREBF-1 associated with EC, including 3 new SNPs. Fourteen percent of EC showed the rs2297508 SNP with C allele, while only 7% had the C allele was present in benign controls (p = 0.027, OR = 1.983). Additionally, the C allele was associated with cancer differentiation (p<0.05) and the depth of myometrial invasion (p<0.05).

**Conclusion:**

Our study indicates that SNP (rs2297508) of SREBF-1 may serve as a genetic predisposition factor for the development of EC and screening of such genetic marker may be helpful in its early detection.

## Introduction

Endometrial cancer (EC) is the most common gynecological malignancy in the western world and the fourth most common cancer in women. The incidence of the EC has increased by 21% since 2008, and the mortality rate has increased significantly over the past two decades [1]. The American Cancer Society estimated 49,500 new cases and 8,200 mortalities in 2013 [2]. The etiology of EC is multifactorial and involves increased exposure to estrogen [3] and genetic risk factors. So far, it has been proven that several SNPs are associated with an increased risk of EC, such as nucleoside diphosphate kinase 1(nm23-H1;rs16949649 and rs2302254), serpin peptidase inhibitor, clade E(PAI-1;rs1799889) and progesterone receptor (PGR;rs11224561) [4]–[6]. To understand the genetic risk factors comprehensively and find effective targets, it is necessary to identify the related SNPs.

Sterol regulatory element-binding proteins (SREBPs) are transcription factors of the helix-loop-helix–leucine zipper (HLH-LZ) family. Three isoforms of SREBP have been identified in mammalian cells: SREBP-1a, SREBP-1c and SREBP-2 [7]. Two genes(SREBF-1 and SREBF-2) are responsible for expressing these proteins of which SREBF-1 gene is located on chromosome 17 p11.2. There is an overlap in the pathways and functions among the individual SREBPs, but most studies suggest that SREBP-1 mainly regulates fatty acid metabolism and SREBP-2 is the main regulator of cholesterol metabolism [8]. SREBP-1 regulates lipid homeostasis by controlling the expression of the key rate-limiting enzymes required for cholesterol and fatty acid synthesis [9]. Aberrant lipogenesis is an important metabolic feature involved in rapid proliferation of the malignant tumor cells. Several studies have shown that tumor cells are capable of reactivating de novo lipid synthesis and expressing elevated levels of fatty acid synthase (FASN) [10] which is regulated by SREBP-1 [11]. Consistent with these findings, SREBP-1 has been demonstrated to have an association with many other malignant tumors such as breast cancer, prostate cancer and colorectal cancer as well [12]–[15].

Through the mechanism of lipid biosynthesis, SREBP-1 has been proposed to be a causative factor of obesity [16]. Obesity on the other hand is considered responsible for various mechanisms that precipitate into carcinogenesis [7]. Through the mechanism of insulin resistance, obesity induces the secretion of insulin from the pancreatic cells. Insulin in turn has a stimulatory effect on SREBP1 [17]. Recently, higher level of SREBP-1 has been detected in EC cells compared to the normal endometrium, and which was more prominent in higher-grade EC. Additionally, knockdown of SREBP1 was found to effectively repress the proliferating capacity of EC cells and tumor growth in vitro, further indicating that SREBP-1 plays an important role in the progression of EC [18].

To our knowledge, no study to date has reported the potential association of SREBF-1 genetic polymorphism with the risk of EC. We hypothesize that SNPs in SREBF-1 might be associated with increased risk of EC, along with several clinical criteria like pathologic grade, clinical stage, pathologic type etc. The SNPs of SREBF-1 may serve as genetic predisposition factors for the development of EC and screening of such genetic markers could be of great value for an earlier detection of this disease.

## Materials and Methods

### Ethics statement

The endorsed written informed consents were acquired from all the participants and the samples were collected with the approval of the Ethics Committee at Qilu Hospital of Shandong University.

In our study, we conducted a case-control analysis with a total of 139 cases and 129 controls. We firstly screened the entire gene of SREBF-1 in 30 unrelated EC patients and 6 controls to identify the associated SNPs. Based on the results we obtained, we chose one SNP (rs2297508) which was found in eight out of the thirty patients and none of the six controls and conducted a case-control study in order to verify its association with EC.

### Study subjects

A total of 268 unbiased candidates were recruited stochastically. The samples of 139 EC specimen and 129 benign controls were collected based on their pathological reports. The endometrial samples from 139 cancer patients were obtained through surgical resection after hysterectomy performed at Qilu hospital Dept. of gynecology. The tissue samples for controls were collected from the patients with no fertility problems and with benign pathologies like leiyomyomas, adenomyosis etc. The individual samples were examined under the microscope for pathological diagnosis for both groups. The EC patients were divided into two groups, 91 without diabetes and obesity and 48 with diabetes and/or obesity and this information was got from the hospital records. The normal controls with diabetes or obesity (BMI≧28 Kg/m^2^) were excluded due to the possible association of SNP (rs2297508) with both of the metabolic disorders as described in the study by Liu J. X.et al.[20]. Also, the controls had no known history of diabetes, obesity or any other malignancies in their family. The mean age of 48 patients with obesity and/or diabetes was 53.09±6.7 and that of the 91 patients without diabetes or obesity was 54.36±9.7(P = 0.196). The mean age of the normal controls (52.46±7.7) was comparable to that of the 91 patients (P = 0.215). The EC patients were treated surgically in Qilu Hospital from 2008 to 2012. They were categorized into endometrioid (type I) and non-endometrioid (type II) EC and the tumors were staged in accordance to the 2009 International Federation of Gynecology and Obstetrics (FIGO) classification.

### DNA preparation

The fresh tissue specimen collected was stored at −80°C refrigeration. Genomic DNA was extracted from the tissues using a QIAamp DNA Mini kit (Qiagen, USA) following the manufacturer's protocol. The extracted DNA was dissolved in TE buffer [10 mMTris (pH 7.8), 1 mM EDTA] and then the concentration was measured with a reference to OD value of 260 nm (BIO-RAD SmartSpec Plus). The final preparation was stored at −20°C for PCR amplification. Amplifications was conducted using a 5 min initial denaturation at 94°C, followed by 30 cycles each lasting 30 sec at 94°C, 30 sec at 60°C,30 sec at 72°C and a 10-min final extension at 72°C. The forward and reverse primers were 5′ GACCTGAGGCTCCTGTGCTAC 3′ and 5′ AAGTCAGTCCATCCTCCCGT 3′ respectively.

### Selection of SREBF-1 polymorphism points

In order to access adequate information about the potential association of SNPs in SREBF-1 with endometrial cancer, we first screened the entire gene of SREBF-1 in 30 unrelated patients and 6 controls by high throughput sequencing technique using PSTAR-II plus (IDN01-M-P2). The 36 subjects were randomly chosen and matched on the basis of their ages. We were able to identify 10 SNPs, including three newly detected ones. All the findings were assayed by Pstar-II 6.0.4 build3 software. For the association study, we selected only one SNP (rs2297508) based on its allelic frequency which exhibited an obvious and significant relation to EC.

### SREBF-1 genotyping

Real-time polymerase chain reaction analysis was used to genotype the SNP (rs2297508) in SREBF-1. The primer sequences for the SNP were as follows: gF 5′ CTCCCCCAGCACCTACGG 3′, cF 5′ CTCCCCCAGCACCTACGC 3′ as the forward primer and R 5′ CTCCCCACTCCTCCCACTAAC 3′ as the reverse primer. The reaction system (20 ul) for rt-PCR included 10 ul All-In-One qPCR Mix, 0.4 ul forward Primer F (2 µM), 0.4 ul reverse Primer R (2 µM), 2 ul genomic DNA and 7.2 ul ddH2O. The reaction procedure was conducted on a real time PCR instrument(Step One Plus(ABI)) dividing into three stages: holding stage at 95°C for 20 s, cycling stage of 40 cycles each including 3 s at 95°C and then 30 s at 60°C, melting curve stage at 60°C for 60 s followed by 95°c for 15 s.

### Statistical Analysis

Hardy–Weinberg equilibrium analyses were conducted to compare genotype frequencies using χ^2^ test among controls (χ^2^ = 1.24, p = 0.26). Comparison of genotype frequencies between EC patients and controls were performed using Pearson χ^2^ test. We also evaluated the relationship between genotype distribution of SREBF-1 SNPs and the clinical criteria of EC (divided on the basis of presence or absence of diabetes and obesity, age, histological type, pathological grading and clinical stages). Analyses were conducted by using the computer software SPSS (version 17.0). P<0.05 was considered the level of statistical significance.

## Results

### SNP identification and genotyping

**Figure 1:**
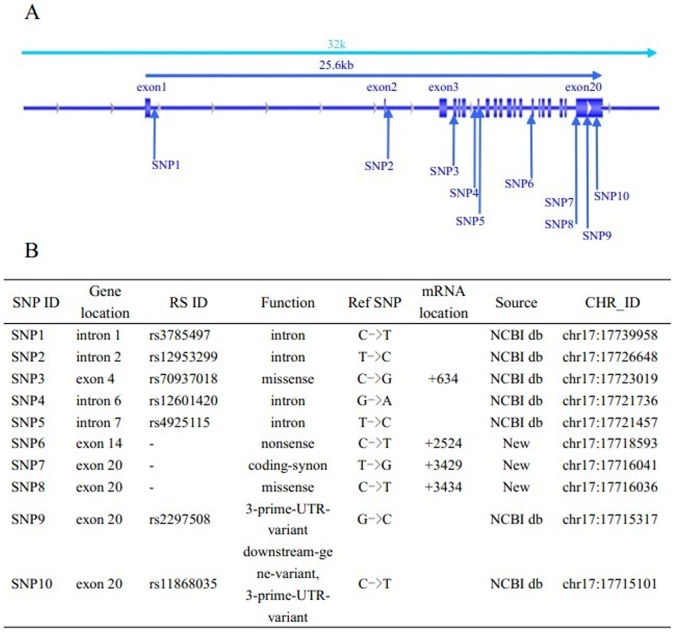
A: SNPs in SREBF-1 gene. The whole SREBF-1gene includes 22 exons. Ten SNPs (SNP1 to SNP10) were identified within the SREBF-1 in our study. FIG. 1 B: SNP ID. SNP1 to SNP10 are listed from 5′-3′ of the SREBF-1. “CHR-ID” shows chromosome ID of ten SNPs in SREBF-1. Listed “RS ID” has been reported in ucsc or NCBI website, “-” expresses newly detected SNP. “Function” column listed altered genetically coded function of the SNPs. “Ref SNP” shows SNP form in original reference sequence. In “mRNA location”, corresponding mRNA positions are showed in consistent with each SNP. (For details:Sequence IDs included in CCDS 32583.1). NCBI db is available from http://www.ncbi.nlm.nih.gov/SNP.

**Figure 2:**
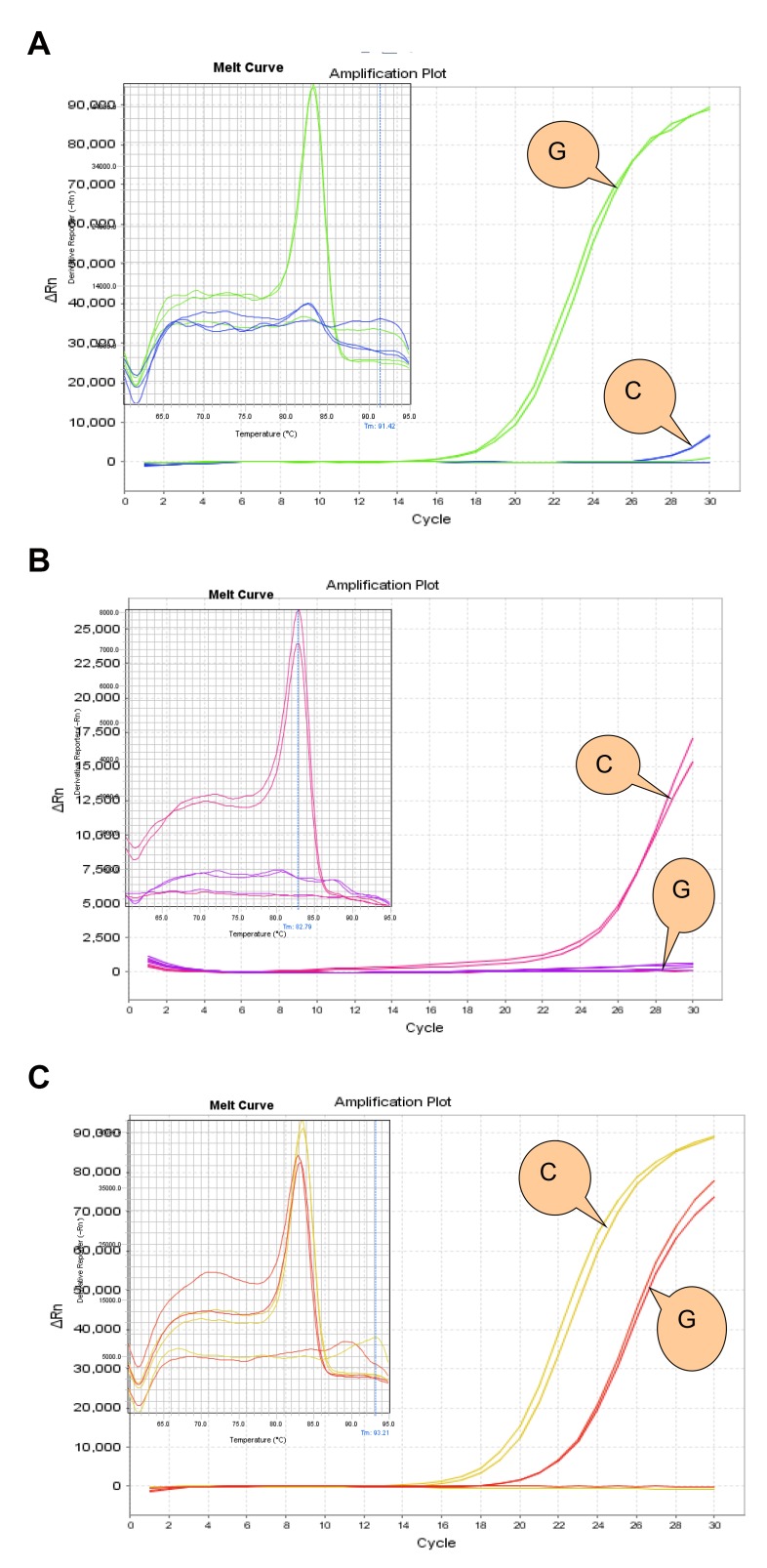
GG, CC and GC genotypes in the samples. Fig. 2 A, B and C show GG, CC and GC genotype respectively as shown by the results obtained by RT-PCR.

Our screening identified 10 polymorphisms (SNP1 to SNP10) including 3 newly detected sites (Fig. 1). The C allele in SNP9 (rs2297508) was detected in eight of thirty patients (26.7%), but in none of the controls. Other detected SNPs showed little difference between patients and controls. (Table 1) Then, we selected SNP9 (rs2297508) for further study and analyzed all experimental samples and controls using real-time polymerase chain reaction. The results (GG, GC or CC) were analyzed by Step One Software V2.1. Genotyping was conducted based on the differing amplification curves observed for different genotypes (Fig. 2).

**Table 1:** Genotypic and allelic frequencies of the 10 SNPs in the 6 controls and 30 EC patients.

Normal controls							EC patients				
Genotype frequency(%)					Allele frequency(%)		Genotype frequency(%)			Allele frequency(%)	
1/1			1/2	2/2	1	2	1/1	1/2	2/2	1	2
SNP1	C/T	83.3	16.7	0	91.7	8.3	93.3	6.7	0	96.7	3.3
SNP2	T/C	100	0	0	100	0	90.0	10.0	0	95.0	5.0
SNP3	C/G	100	0	0	100	0	96.7	3.3	0	98.3	1.7
SNP4	G/A	0	66.7	33.3	33.3	66.7	3.3	56.7	40.0	31.7	68.3
SNP5	T/C	100	0	0	100	0	93.4	3.3	3.3	90.0	10.0
SNP6	C/T	100	0	0	100	0	96.7	3.3	0	98.3	1.7
SNP7	T/G	100	0	0	100	0	96.7	3.3	0	98.3	1.7
SNP8	C/T	100	0	0	100	0	96.7	3.3	0	98.3	1.7
SNP9	G/C	100	0	0	100	0	73.3	20.0	6.7	83.3	16.7
SNP10	C/T	0	16.7	83.3	8.3	91.7	6.7	16.7	76.6	15.0	85.0

### Association between SNP rs2297508 and risk of EC

The allele distribution of SNP (rs2297508) on SREBF-1 genotypes in control groups is consistent with Hardy-Weinberg equilibrium as tested by χ^2^ test (χ^2^ = 2.27, P = 0.13). The variation in the distribution of C allele between EC patients and normal controls was clearly shown (P = 0.027). The χ^2^ test revealed that the GC/CC genotypes or C allele carriers had an increased risk of EC (OR = 1.966 and OR = 1.983 respectively) compared to GG or G carriers respectively. Confidence intervals of the odds ratio (OR) was used to describe the limitations within which the estimated calculations should be valid. The comparison of genotypic and allelic frequencies of the SNP between EC patients and normal controls is shown in Table 2. The results were consistent with our preliminary high throughput experiment.

**Table 2:** Genotype/allele frequency of SREBF-1(rs2297508) in EC patients and normal controls.

SREBF-1	Benign	EC patients(91)	?^2^	P-value	OR	95%CI
(rs2297508)	Controls(129)					
GG	111(86.0%)	69(75.8%)			1	
GC/CC	18(14.0%)	22(24.2%)	3.748	0.053	1.966	0.985–3.926
G	238(92.25%)	156(85.7%)			1	
C	20(7.75%)	26(14.2%)	4.867	0.027	1.983	1.070–3.676

OR odds ratio Cl confidence limit.

### Association between SNP rs2297508 and pathological grade and the depth of myometrial invasion of EC

We have attempted to explain the role of polymorphic locus rs2297508 on co-existing conditions like diabetes, obesity, as well as on different age groups, pathological types and pathological grades of the cancer, clinical stages and myometrial invasion in EC patients (Table 3). Since diabetes and obesity are two major risks known to be closely tied to EC, and the SNP being a crucial marker of the genetic predisposition of EC, it could be assumed that there was a potential effect of this particular polymorphism on the condition. We divided the EC patients into two groups (with diabetes or obesity and without). However, no significant difference in allele frequency was evident between the two. Other criteria for dividing the subjects into various groups were determined according to previous reports as well as clinical significance [4], [5]. The results showed that the SNP C variant carriers had higher pathological grade and deeper myometrial invasion (OR = 2.042 and OR = 2.233 respectively) compared to G carriers. Furthermore, the GC/CC genotype and the C allele were significantly associated with pathological grade (χ^2^ = 4.095, P = 0.043 and χ^2^ = 3.893, P = 0.048 respectively) and myometrial invasion (χ^2^ = 4.018, P = 0.045 and χ^2^ = 4.170, P = 0.041 respectively). Other criteria such as age, pathological type and clinical stage did not show any significant association with the SNP.

**Table 3:** Genotype/allele distribution of SREBF-1(rs2297508) in EC patients with different characteristics.

Endometrial cancer(n = 139)		Rs2297508				Rs2297508			
		GG	GC/CC^a^	?^2^	P-value	G	C^b^	?^2^	P-value
Diabetes and obesity condition	None	69(75.8%)	22(24.2%)			156(85.7%)	26(14.3%)		
	With one of the two or both	36(75.0%)	12(25.0%)	0.012	0.914	81(84.4%)	15(15.6%)	0.765	0.090
OR		1.045				1.111			
95%CI		0.465–2.352				0.557–2.215			
Age (year)	≤50	31(72.1%)	12(27.9%)			72(83.7%)	14(16.3%)		
	>50	74(77.1%)	22(22.9%)	0.400	0.527	165(85.9%)	27(14.1%)	0.232	0.630
OR		0.768				0.842			
95%CI		0.339–1.742				0.417–1.699			
Pathologic types	Endometrioid	88(75.2%)	29(24.8%)			198(84.6%)	36(15.4%)		
	Non-endometrioid	17(77.3%)	5(22.7%)	0.042	0.837	39(88.6%)	5(11.4%)	0.476	0.490
OR		0.892				0.705			
95%CI		0.303–2.633				0.260–1.910			
Pathological grade	Low grade	85(81.7%)	19(18.3%)			189(87.5%)	27(12.5%)		
	High grade	20(64.5%)	11(35.5%)	4.095	0.043	48(77.4%)	14(22.6%)	3.893	0.048
OR		2.461				2.042			
95%CI		1.012–5.980				0.995–4.190			
Clinical Stages	I	75(72.8%)	28(27.2%)			173(84.0%)	33(16.0%)		
	II	14(87.5%)	2(12.5%)	1.584^c^	0.208^c^	29(90.6%)	3(9.4%)	0.319^c^	0.572^c^
	III–IV	16(80.0%)	4(20.0%)	0.449^d^	0.503^d^	35(87.5%)	5(12.5%)	0.007^d^	0.935^d^
OR^c^		0.383				0.693			
95%CI^c^		0.082–1.792				0.193–2.488			
OR^d^		0.670				0.957			
95%CI^d^		0.206–2.176				0.336–2.730			
myometrial invasion	<1/2	60(77.9%)	17(22.1%)			134(87.0%)	20(13.0%)		
	≥1/2	15(57.7%)	11(42.3%)	4.018	0.045	39(75.0%)	13(25.0%)	4.170	0.041
OR		2.588				2.233			
95%CI		1.005–6.667				1.020–4.892			

Statistical analysis:Pearson χ2 test.

Age ≤50 years old, endometrioid type, low grade, stage I and myometrial invasion <1/2 were considered as references for comparison.

a Odds ratio(OR) of the GC/CC against the GG genotypes.

b Odds ratio(OR) of the C against the G alleles.

c Comparison between stage I and II.

d Comparison between stage I and III–IV.

## Discussion

Lipid biosynthesis is essential for the maintenance of cellular homeostasis while increased de novo lipid synthesis is a common metabolic feature of carcinogenesis [19]. The increased expression of FASN and LDLR (Low Density Lipoprotein Receptor) in tumor cells attest to this statement [20]. SREBPs can regulate various enzymes involved in fatty-acid and cholesterol biosynthesis. Apart from influencing several enzymes involved in lipogenesis, SREBP also participates in the conversion of androgen into estrogen through the aromatization reaction thus raising the level of estrogen in the circulation. Obesity has also been found responsible for lowering the levels of progesterone which has a strong antagonizing effect on the carcinogenic function of estrogen [21]. Moreover, SREBP1 is also associated with modulating the transcription of the enzyme 17β-Hydroxysteroid dehydrogenase type 12 (17 β -HSD12) which is responsible for the transformation of estrone (E1) to a more potent form, estradiol (E2) [22], [23]. So, we can deduce that SREBP activity is required for tumor growth, implying that SREBP plays a significant role in oncogenesis. In addition, the aberrantly increased expression of SREBP-1 has been found in several cancers, including EC [18].

In this research we postulated that SNPs in SREBF-1gene have potential role in genetic predisposition of EC. A previous study detected 19 polymorphisms in SREBF-1 and demonstrated the association between SREBF-1 polymorphisms and obesity as well as type II diabetes [19]. Furthermore, epidemiological studies have confirmed that obesity is one of the major risk factors of EC while SREBF-1 gene polymorphisms have associations with metabolic diseases (type2 diabetes and obesity) [24]. Another study concluded that the SNPs rs2297508 and rs11868035 in the SREBP-1c gene have relationships with increased risk of T2DM and dyslipidemia in the Chinese population. The same study also showed that the SNP (rs11868035) is significantly associated with insulin resistance (IR) in diabetic patients [25]. However, the studies, demonstrating the relationship between SREBF-1 genetic polymorphisms and cancers, are rare. Up till now, only Daniele Campa and his co-workers have investigated the relationship between SNPs in SREBF-1 and oncogenesis of breast cancer, but no statistically significant associations were found between the SNPs detected and the risk of breast cancer [26]. Thus we have also further assessed our hypothesis that the genetic polymorphisms are linked with diabetes and obesity along with few other clinical criteria associated with EC.

To our knowledge, this is the first report examining the role of SREBF-1 gene polymorphisms in EC. In our study, we found that the distribution of the SNP (rs2297508) had a distinct demarcation between EC patients and benign controls. The C allele in the SNP was associated with higher susceptibility to EC. Therefore, we predict the high possibility of C allele in the SNP having a crucial relationship with the risk of EC and we believe that further exploration would lead researchers towards developing a new therapeutic target for the treatment of EC.

We further analyzed the association of different clinical characteristics in EC patients with the SNP. First, we evaluated association between diabetes and/or obesity and genotypic or allelic frequency in EC patients. But no statistical significance was found between the two groups. That is to say, the SNP has the same distribution in EC patients irrespective of the presence or the absence of risk factors like diabetes and obesity. It suggested that there was abnormal lipid metabolism associated with the SNP in EC which is independent of diabetes and obesity. Then we compared age criteria, pathological types and clinical stages, but no significant associations were obtained, thus providing evidence that there exists no association between the SNP and those clinical characteristics. Consistence with the report presented by Li W. et al. [21], the C allele in the SNP exhibited a significant association with higher-grade tumor. Moreover, the patients with C allele showed a higher risk of developing a deeper myometrial invasion as compared to those with G allele at the SNP locus. This finding might result from late detection of the disease however we cannot exclude the possibility of the influence of SNP. There might be a potential relationship between pathological grade of tumor as well as depth of myometrial invasion. However further research is needed to clarify this concept. In summary, the C allele seems to be a marker for a higher grade tumor and deeper myometrial invasion in EC, and hence could act as an indispensable marker to be taken into account in clinical evaluation and treatment of EC in the near future.

In conclusion, our study mainly investigated the association between the SNP (rs2297508) in SREBF-1 and the risk of EC and found that C allele of the SNP is potentially a risk factor for EC. At the same time, we analyzed the association of the SNP with different clinical criteria of EC and the results showed that patients with higher C allelic frequency were more susceptible to develop high grade tumor and deep myometrial invasion. The results indicated that the SREBF-1 polymorphism might play an essential role in increasing the genetic susceptibility to EC. Therefore we assume that this could assist the physician in clinical diagnosis of the condition and would also guide towards a targeted clinical therapy.

In our study, we employed high throughput sequencing, a reliable technique with great accuracy and precision, to detect 10 SNPs associated with EC including three new ones which might be of significance for other studies in the future. Furthermore, we confirmed that the SNP (rs2297508) in SREBF-1 has a significant influence on EC susceptibility and clinical criteria. However, our study has some limitations. At the subject level, we don't have lifestyle data such as dietary intake, physical activity, hormonal contraceptive use etc. that are likely to influence the susceptibility of the SNPs to EC risk. Further, it is unknown whether the SNP works in coordination with other SNPs in increasing the risk of EC. Future studies of larger sample size will be necessary to study the influence of these factors on the association between the SNP and risk of EC.

## Supporting Information

Information S1 (DOC)
